# Using verbal and social autopsies to explore health-seeking behaviour among HIV-positive women in Kenya: a retrospective study

**DOI:** 10.1186/1472-6874-14-77

**Published:** 2014-06-27

**Authors:** Rebecca Njuki, James Kimani, Francis Obare, Charlotte Warren

**Affiliations:** 1 Center for Population Health Research Management, Magharibi Place, 2nd Floor, Room 2, P.O. Box 19607–00202, Nairobi, Kenya; 2 Population Council, General Accident Insurance House, Ralph Bunche Road, P.O. Box 17643–00500, Nairobi, Kenya

**Keywords:** Verbal and social autopsy, HIV, Health care seeking behaviour, Women, Kenya

## Abstract

**Background:**

There is limited understanding of the factors that influence decisions to seek HIV care and treatment services in community settings. The aim of this study was to explore the socio-cultural and health system factors affecting health-seeking behaviour among deceased women in Kenya who were living with HIV at the time of death.

**Methods:**

Out of a total of 796 deaths for which a caregiver was available to provide information, retrospective data were drawn from verbal and social autopsies administered to caregivers of 218 women who had died of AIDS-related illnesses aged 15 to 49 years. Information was collected on essential elements of the care-seeking process from the onset of severe illness episodes and analysed using qualitative and quantitative techniques.

**Results:**

Results from the quantitative data showed that poor women were less likely to access formal health services (OR = 0.2; p < 0.001) compared to non-poor women. The qualitative data showed that socioeconomic status, poor knowledge and understanding of AIDS-related illness, distance to facility and transportation costs, medical pluralism, stigma, low HIV risk perception, lack of family support and health care system barriers contributed to delays/constraints in seeking care.

**Conclusions:**

The findings highlight important issues that have implications for addressing challenges faced by women living with HIV, including non-adherence to treatment regimen and late diagnosis of HIV. Provision of transportation subsidies as part of the national social safety-net strategy can help in addressing financial constraints associated with transportation costs among poor women living with HIV.

## Background

At the end of 2011, an estimated 34 million people were living with HIV globally with sub-Saharan Africa (SSA) being disproportionately affected by the epidemic [1]. According to UNAIDS, the region accounts for about 68% of all people living with HIV worldwide and women are mostly affected accounting for over half (59%) of all people living with HIV [2]. In Kenya, according to the 2008–2009 Kenya Demographic and Health Survey (KDHS), the national HIV prevalence among adults aged 15–49 years was 6.3% [3]. HIV prevalence was twice as high among women compared to men (8% among women and 4% among men). In addition, HIV prevalence was higher in the urban (6.5%) than in the rural (5.1%) areas [4]. Heterosexual intercourse is the predominant mode of HIV transmission in the country with 44% of new infections occurring among regular or steady partners while sex between casual sexual partners account for 20% [5]. Expansion of access to life-saving antiretroviral therapy (ART) in sub-Saharan Africa has enabled people to lead productive lives. In Kenya, the number of HIV-positive individuals accessing antiretroviral therapy increased from less than 10,000 in 2003 to over 430,000 in 2010 [6]. In addition in all public facilities in Kenya, HIV care and treatment is provided to all those who need the services at no cost to the clients.

Evidence on health-seeking behaviour among people living with HIV has primarily focused on their HIV treatment experience in clinical settings [7,8], on demonstrating the feasibility of HIV treatment in such settings [9-11], monitoring how treatment is delivered and individuals’ treatment adherence [12]. A number of factors have been shown to influence health-seeking behaviour of people seeking HIV treatment and care. Previous studies have demonstrated that physical distance and accessibility of services, availability and quality of services, costs related to services and transportation, availability of commodities and supplies, negative attitudes among health workers, fear of stigma and discrimination, low HIV risk perception and socioeconomic status are important factors that determine utilization of HIV treatment and care services [13-23]. Among people living with HIV, available evidence shows that they seek care from formal (such as government and private health facilities) and informal sources (such as traditional healers, home and shops) [24].

Socio-economic status is closely linked to vulnerability to HIV infection and access to health services and information: women who have higher levels of wealth and education are much more likely to use contraceptives, have knowledge regarding HIV prevention and access treatment and care services [3]. Although poverty and socio-economic inequality affect both women and men, women’s subordinate status in patriarchal societies implies that they have less access to education and paid employment besides lacking control over financial and productive resources [3].

There has been a growing need to reduce mortality related to HIV for women and, therefore, the need to develop health services for women living with HIV to encourage them to seek help early. However, there has been little emphasis on understanding the factors that influence decisions to seek HIV care and treatment services in community settings and the barriers to health seeking among these women. The objective of this paper is to explore the socio-cultural and health system factors affecting health-seeking behaviour among women living with HIV (WLHIV) in Kenya. The study focuses on the health and care seeking behaviour of deceased women from the time they started suffering from severe illness episodes to death.

## Methods

The study that provided the data for this paper was part of a project implemented by the Population Council which aimed at evaluating the effect of the output-based aid (OBA) voucher program (being implemented in six districts in the country) on reproductive health behaviours and outcomes [25]. The objective of the voucher program is to significantly reduce maternal and neonatal mortality by increasing the number of health facility deliveries and improving access to appropriate health services—including reproductive health—for the poor through incentives for increased demand and improved service provision. Death audits were conducted to determine the prevalence of maternal and newborn mortality and understand health-seeking behaviour prior to the occurrence of deaths in the OBA study sites. Data were collected between June and December 2010 and involved a cross-sectional survey of deaths of women aged 15–49 years living within a 5 km radius of accredited OBA facilities in three districts (Kisumu, Kiambu and Kitui). The primary sampling unit for the verbal autopsy was the sub-location. In each district, 14 sub-locations were randomly selected from among those within the stipulated distance (within 5 km radius) to accredited facilities. Three enumeration areas/villages were then randomly selected from each of the sampled sub-locations. In the selected enumeration areas, all deaths that occurred among women aged 15–49 years were identified through the death notification register that is maintained by the local government administration. In Kenya, the civil registration process includes a chief (who heads a location) who is tasked with registering all deaths occurring in that particular location. The chiefs are supported by assistant chiefs and village elders to ensure all deaths are registered and a death notification card is issued as a requirement for acquiring a burial permit.

To collect the retrospective data, we used verbal and social autopsies. The World Health Organization (WHO) standard verbal autopsy questionnaires and a social autopsy tool were administered to caregivers of women aged 15–49 years who had died between 1996 and 2010. Only one caregiver was interviewed for each death. Verbal autopsy is a research method that helps determine probable causes of death in cases where there was no medical record or formal medical attention given and involves conducting interviews with next of kin or other caregivers [26]. Social autopsy, on the other hand, refers to an interview process with a next of kin or other caregivers and aims at identifying social, behavioural, and health systems contributors to deaths in the community [27]. Both verbal and social autopsies provide evidence for informing health care programmers and policymakers in designing and implementing initiatives for improving maternal and child health [27]. A total of 819 deaths were identified. However, because some caregivers could not be traced, our analyses focused on 796 deaths where verbal and social autopsy interviews were conducted with an immediate caregiver of the deceased. Interviews were conducted in Kiswahili or a local language. The caregiver was selected by the family members on the basis of her/his participation in the health and care seeking process for the deceased.

All the 796 deaths were coded by two trained medical doctors to determine the causes of death using the International Classification of Disease (ICD)-10. Where the doctors did not agree on the causes of death, they met to arrive at a consensus, and if they still did not agree after the meeting, the cause of death was recorded as unknown. Of the 796 deaths, 218 were coded as HIV/AIDS-related deaths. It is worth noting that a total of 64 deaths met the World Health Organization (WHO) definition of maternal death due to direct or indirect causes. Out of the 64 maternal deaths, 11 (17%) were caused by AIDS-related complications. Overall, AIDS-related complications accounted for nearly a third of the maternal deaths.

Information was collected on a wide range of issues, including socio-demographic characteristics, essential elements of the care-seeking process such as recognition of the symptoms and signs associated with illness, whether adequate care was provided, whether and what type of outside-the-home care was sought (informal, formal, or both), cause and place of death. Social autopsy interviews explored health and care-seeking behaviour of the deceased including any preventive care received, the diagnostic procedures followed, the type and timing of any treatment provided within or outside the home, any barriers/delays encountered during care seeking, and the quality of health care provided (from the client’s perspective).

Data analysis was conducted separately for the verbal and social autopsy. For quantitative data derived from the verbal autopsy, descriptive statistics were used to characterize the study sample, including primary caregiver’s relationship, place where care was sought, number of contacts with formal health services, place of death, household socioeconomic status, marital status, education and age. Quantitative data were analysed using STATA® version 10. Cross-tabulations with Chi-square and Fisher’s exact tests were done to test the associations between key variables of interest. Multivariable logistic regression analysis was conducted to examine the relationship between having contact with formal health services and socioeconomic status after controlling for confounding factors. Regression analysis was also performed to examine the relationship between the use of more than one source of medical care (medical pluralism) and socioeconomic status. Socioeconomic status was assessed using a poverty grading tool that consisted of eight items on household assets and amenities, expenditure or income, and access to health services customized to each district to identify OBA voucher clients from the community. For each item, the score ranged from 1 to 3 with the maximum score for all the items being 24. Women scoring between eight (which is the minimum) and 16 points on the poverty grading tool were deemed poor.

Qualitative interviews were tape recorded, transcribed verbatim and translated into English. There was, however, no back-translation due to financial constraints. We adopted the grounded theory methods in data analysis which involved both inductive and deductive approaches. Analysis was done by two researchers to ensure reliability in the coding and results. Following coding, a full list of themes was available for categorization within a hierarchical framework of main and sub-themes. The thematic framework was then systematically applied to all of the interview transcripts. We looked for patterns and associations of the themes and compared and contrasted within and between the different regions and age groups. The qualitative transcripts were managed and analysed using QSR NVivo 9 Software © (International Pty 2007, Australia). Analysis of the data was guided by the pathway to survival model [28,29] and the three-delay framework [30].

### Ethical considerations

Written informed consent for participation in the study was obtained from informants. Ethical and research clearance was obtained from the Institutional Review Board of the Population Council, the Ethics Review Committee of the Kenya Medical Research Institute (KEMRI), the National Council for Science and Technology (NCST), and the Ministries of Health (Public Health and Medical Services).

## Results

### Quantitative results

**Figure 1:**
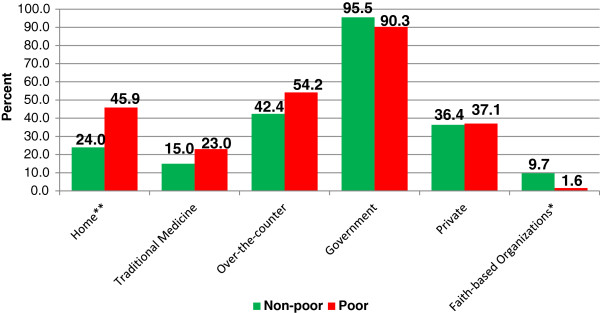
**Type of care sought by socioeconomic status.** ** p<0.01; * p<0.05.

The background characteristics of the study sample are described in Table 1. The median age of the women at the time of death was 34 years (results not shown). The majority of them had primary or lower levels of education, were divorced, separated or widowed, and were from non-poor households. In addition, for the majority of the women, information was provided by their parents. With respect to access to medical care, a similar proportion of women sought care from over-the-counter (pharmacy or drug stores) and government health facilities while more than a third of the women sought care from private health facilities. Nearly two-thirds of the women had made contact with formal health services one or two times a month prior to death, while most died at home.The results from bivariate analysis examining the association between the place where a woman sought treatment and socioeconomic status (poor versus non-poor) are presented in Figure 1. A higher proportion of women from poor households compared to those from non-poor households sought care from informal sources, including over-the-counter (54.2% versus 42.4%; p = 0.121), home (45.9% versus 24.0%; p = 0.002) and traditional medicine (23.0% versus 15.0%; p = 0.167); however, the differences were not statistically significant for over-the-counter and traditional medicine. Majority of the women from poor and non-poor households sought care from government health facilities (95.5% and 90.3%, respectively; p = 0.151). Besides, a similar proportion of women from both socio-economic groups sought care from private health facilities. A significantly higher proportion of women from non-poor households compared to those from poor households (9.7% versus 1.6%; p = 0.044) sought care from health facilities managed by faith-based organizations (FBO). Further analysis was also conducted to examine the association between socioeconomic status and whether a woman had any contact with formal health services one month prior to death. On average, non-poor women had contact with formal health services 2.3 times (standard deviation = 3.6) compared to 1.8 times (standard deviation = 2.1) among poor women (p = 0.383). A higher proportion of poor women did not have any contact with formal health services compared to their non-poor counterparts in the month before death (22.4% versus 6.3%; p = 0.001). A slightly higher proportion of poor women compared to non-poor women (76% versus 69%; p = 0.308) sought care from more than one place (medical pluralism).

**Table 1:** Background characteristics of study sample, access to health services and place of death

**Variable**	**Women (N = 218)**	
	**N**	**%**
**Age**		
19–29 years	67	31.0
30–39 years	103	47.7
40+ years	46	21.3
**Education**		
Primary and below	166	77.9
Secondary and higher	47	22.1
**Marital status**		
Single	66	30.3
Married	57	26.2
Divorced/separated/widowed	95	43.6
**Household socioeconomic status**		
Poor	62	28.7
Non-poor	154	71.3
**Relationship of primary caregiver**		
Parent	109	50.0
Spouse	27	12.4
Brother/sister	54	24.8
Children	12	5.5
Other relative	11	5.1
Not related	5	2.3
**Place where care was sought**^**a**^		
Home	66	30.4
Traditional healer	37	17.1
Over-the-counter	97	45.8
Government health facility	96	45.3
Private health facility	80	36.7
Faith-based facility	16	7.3
**Number of contacts with formal health services a month prior to death**		
None	22	10.8
1–2 times	132	65.0
3 and above times	49	24.1
**Place of death**		
Home	130	60.5
Health facility	80	37.2
Other place	5	2.3
**Year of death**		
1996-2000	4	1.9
2001-2005	64	30.5
2006-2010	142	67.6

Results from the multivariable logistic regression analysis, predicting the likelihood of seeking formal health care show that poor women were significantly less likely to have contact with formal health services (OR = 0.2; p < 0.001; 95% CI, 0.1-0.5) compared to those from non-poor households (Table 2). Education, marital status and age were not significantly associated with contact with formal health services.

**Table 2:** Crude and Adjusted odds ratios from multivariate logistic regression model predicting contact with formal health services among women who had died of AIDS-related illnesses

**Variables**			**Contact with formal health services (N = 196)**	
	**Crude ORs**	**95% CI**	**Adjusted ORs**	**95% CI**
**Poverty status***(Ref = non-poor)*				
Poor	0.2**	[0.1 – 0.6]	0.2***	[0.1 – 0.5]
**Age of deceased women***(Ref = 19–29 years)*				
30–39 years	1.1	[0.4 – 2.8]	1.0	[0.3 – 2.8]
40+ years	3.1	[0.6 – 15.4]	3.9	[0.7 – 21.8]
**Marital status***(Ref = Never married)*				
Married	0.7	[0.2 – 2.5]	0.7	[0.2 – 2.4]
Divorced/separated/widowed	0.6	[0.2 – 1.7]	0.5	[0.2 – 1.8]
**Education***(Ref = Primary and below)*				
Secondary and higher	2.9	[0.6 – 12.8]	2.6	[0.6 – 12.4]

** *p < 0.01*; *** *p < 0.001*; Ref = reference category.

Results from the multivariable logistic regression model predicting the likelihood of seeking care from more than one source are presented in Table 3. It is worth noting that age and marital status were significantly associated with medical pluralism. Specifically, women aged 40 years and above were significantly less likely to seek care from more than one source compared to those aged 19–29 years. Married women were 3.6 times more likely to seek care from more than one place compared to never married women (p < 0.01). Socioeconomic status and education were not significantly associated with medical pluralism.

**Table 3:** Crude and Adjusted odds ratios from multivariate logistic regression model predicting use of more than one source of care among women who had died of AIDS-related illnesses

**Variables**			**Sought care from more than one place (N = 210)**	
	**Crude ORs**	**95% CI**	**Adjusted ORs**	**95% CI**
**Poverty status***(Ref = non-poor)*				
Poor	1.4	[0.7 – 2.8]	1.5	[0.7 – 3.0]
**Age of deceased women***(Ref = 19–29 years)*				
30–39 years	0.6	[0.3 – 1.3]	0.5	[0.3 – 1.2]
40+ years	0.4*	[0.2 – 0.9]	0.3*	[0.1 – 0.8]
**Marital status***(Ref = Never married)*				
Married	3.3**	[1.4 – 7.5]	3.6**	[1.5 – 8.8]
Divorced/separated/widowed	1.7	[0.9 – 3.4]	1.8	[0.9 – 3.7]
**Education***(Ref = Primary and below)*				
Secondary and higher	0.8	[0.4 – 1.5]	0.9	[0.4 – 2.0]

* *p < 0.05*; ** *p < 0.01*; Ref = reference category.

### Qualitative findings

Qualitative findings showed that a number of factors were responsible for delays and constraints to formal health care seeking.

### Poor understanding of HIV-related opportunistic infections

As reported by caregivers, there was poor knowledge and understanding of signs and symptoms of severe illnesses perceived to be associated with HIV/AIDS among the women. Many people interpreted AIDS-related conditions such as headache, stomach ache, chest pains and diarrhoea as malaria or common illnesses, prompting them to seek over-the-counter malaria medications. This often resulted in multiple uses of different types of anti-malaria drugs even in areas with low prevalence of malaria. Due to the perceived poor knowledge and lack of understanding of symptoms associated with HIV-related illnesses, it emerged that in some cases HIV/AIDS was associated with witchcraft and evil spirits. The following quotes provide insights into some of the conditions that were associated with AIDS:

*“The time when she was sick she stayed here at home and we brought for her the drugs. She also could buy drugs from the chemists. We thought it was normal chest complications, but it was the critical chest complications that killed her.”* (Mother to deceased)

*“I went to see her but on asking her what the problem was she told me she had stomach ache and headache. But on telling her that I take her to hospital she refused.”* (Son to deceased)

*“Before she died, I messed up, as I got late in intervening as she had severe diarrhoea and within that week she died, I could have taken her on Monday but I did not think it was serious. I got late and took her to the hospital on a Friday.”* (Sister to deceased)

### Medical pluralism

Caregivers reported that the majority of women living with HIV first opted for home treatment, then purchased over-the-counter drugs, then sought care from a traditional healer, then visited a health facility before going back to a traditional healer. Perception of HIV risk and severity of illnesses determined the type of treatment sought by the women. Women in stable relationships often believed they were not infected and, therefore, sought over-the-counter medication. However, when the illnesses were severe and the other treatment options had failed then the women resorted to seeking treatment at health facilities. HIV-related stigma was also a key factor in the utilization of care and treatment. Home remedies and traditional healers were mostly preferred by the women due to fear of stigmatization. The findings showed that women sought treatment in three ways, namely; (i) going to a witchdoctor or traditional medicine man, (ii) taking home-made remedies believing that it is a mild illness that will go away, and (iii) going to a health facility. The following excerpts highlight some of the ways that women sought care:

*“My wife began to have body pain, then I bought for her medicine from the shop, but she never got better. I took her to xxxx Health Centre where she was treated and we came back home, but for one month, she never got well. She was rushed to xxxx district hospital and given medicine. She came back home and stayed for about 3 months, and she did not get well; the stomach started swelling again. When it was swollen, instead of taking her back to the hospital, we took her to witch doctors. But one of the ladies told me to take her back to the hospital, and I took her back to xxxx district hospital, and she was tested. She came and told me she had been found with a virus.”* (Husband to deceased)

*“You know in this area, when a person becomes sick, our first thought is to see a witchdoctor. So for this illness, we used to mix medical doctor, witch doctor for the first 5 years, but when she went to xxxx Hospital, it was found out she has typhoid.”* (Daughter to deceased)

*“There are the witchdoctors here who cheat people. That is where she was taken. Also she could be brought a witchdoctor at home, who could tell her it is this place that is the cause. Then it was realized that is not the cause.”* (Neighbour to deceased)

### Non-disclosure of HIV status and caregiver role of women

Many caregivers reported that non-disclosure of HIV status was a problem as many of them learned about the women’s status from the health care providers. Besides stigma, non-disclosure of HIV status among women infected with HIV was related to what they perceived as the impact of the illness on their families. Many women did not want to burden their families financially and, therefore, opted not to seek care and treatment despite the fact that some caregivers reported that they suspected the women were HIV-positive. Others were psychologically affected by the likely impact the illness would have on their children and who would care for them when they died. Most caregivers mentioned the fear of the unknown for the children as the main reason women did not disclose their HIV status to their children. Further, women declined hospital admission in order to take care of children.

*“Sometimes I feel that if she was open and had said early enough, she could be alive. This is because we have another cousin who is also HIV positive and goes to hospital at xxx and has given my phone number there, so that if she is in any sort of need and I am contacted, she can be assisted immediately. But xxxx was quiet and did not disclose her problem.”* (Friend to deceased)

*“She went to the hospital and was given drugs to help her. She was also tested for HIV and told she has but she said she does not want to be admitted as she has children who will suffer and the mother is old to take care of the children.”* (Friend to deceased)

*“She was seriously sick but could go to work while sick and after coming home she could tell me that the work was bad until I told her to leave the job and stay with us here at home. She was refusing to go to the hospital because of fear of being tested and to be told her status. But I talked to her until she agreed to go to the hospital four months later.”* (Mother to deceased)

### Access to health services

Distance to facility coupled with transportation costs and challenges emerged as major barriers to accessing care. Caregivers also reported that in some areas only a few health facilities provided HIV treatment. Due to perceived lack of confidentiality in handling client information by facilities that were nearby, women preferred to seek care in health facilities that were further away from their homes.

*I came home and explained to her about the illness, and told her we should begin the clinic, which we began in February 2006, and she even began going for clinics alone, but we felt it was far, because of the bus fare and the bills.”* (Sister to deceased)

*“There are no vehicles in the area so we had to stay home.”* (Mother to deceased)

*“So she told me to take her to the hospital, but insisted I take her to another hospital at xxxx. So we travelled on a Friday, but we found it difficult to get to the facility. It had rained badly, so the place was swampy, and we could not pass since I was using a bicycle to transport her. It was impassable; we discussed and decided to go to another facility xxxx. But we went to this other facility on a Sunday.”* (Brother to deceased)

Health system factors such as poor referral systems, lack of care before referral, perceived lack of providers, lack of equipment/drugs, discrimination by providers, especially towards poor clients, and perceived poor quality of care, were cited as reasons for low utilization of HIV-related health care services as exemplified by the following quotes:

*“When she took the medication, she started having diarrhoea a lot, and we had been told to take her for other tests, but we did not take her. When we went there, we found there was no doctor.”* (Daughter to deceased)

*“She was given Malaria medicines as they did not have equipment to examine her.”* (Daughter to deceased)

*“The patient was referred to xxxx District Hospital because the hospital did not have supply of drugs.”* (Son to deceased)

*“For her the illness bothered her so much because she had no money for her treatment and at the hospital they expected money plus for the drugs. When she went to the hospital she was told to first look for money and then come back. She was ill and could not get the drugs. When she came home she could become sick and got seriously sick.”* (Husband to deceased)

### HIV stigma, denial, and low HIV risk perception

Majority of the caregivers reported that low HIV risk perception in the community was responsible for women’s reluctance to seek HIV testing services. This observation was particularly common among women with only one sexual partner. Another factor that emerged as a barrier to seeking HIV testing was lack of awareness about facilities that can be trusted to maintain confidentiality of a client’s HIV status. Caregivers reported a general fear in the community about a person knowing about their status. Further, women who died while pregnant due to HIV-related complications did not attend antenatal care (ANC) clinic due to perceived fear of ‘coercive’ HIV testing (in Kenya there is an opt out policy regarding testing). According to the caregivers, the women felt that health care providers at the ANC clinics would not maintain confidentiality, hence increasing the risk of exposing them to HIV-related stigma.

*“My friend had a problem of coughing and back ache and her chest had pains so I used to advise her to know her HIV status, I used to tell her ‘the way I see you coughing, your cough is too deep and let me take you to xxxx health facility so that you can know your status maybe its TB’ [tuberculosis] but she was tough headed, she didn’t go. She said she is not ill, that illness is only malaria so I let her be and she got worse, so for some time she avoided me because she knew I would take her there (xxxx health facility). She wanted to go to xxxx and xxxx health facilities and the one who took her is another lady called Florence (fictitious name).”* (Friend to deceased)

*You know the first time she was told she was HIV positive she denied saying she is not positive; she denied up to the last moment when she died. Later there was one hospital sister who informed my brother’s wife that she was HIV positive.”* (Neighbour to deceased)

*“When I took her to xxxx hospital she was counselled. Then I took her for testing. Her blood was tested when I was with her and I was told she is HIV positive. After she was told she is HIV positive, and by then she was severely ill, she was shocked from the time she got to know her status. After being told by the provider, she did not stay for long. I even had to force her to take the medicines, though she would refuse.”* (Daughter to deceased)

*“I told her she was to take the medicine as the times we are in today are bad times, as she is not the only one who is infected with HIV, but others too have HIV. But she still insisted she will not take the medicines. All the same, we took the medicine with us at home but she could take some medicine and hide others. So I spoke with her again, told her ‘you know I am your mother, so instead of dying and leaving your children to die, take this medicine’ but she said she will not take as she knows it’s her husband who has infected her with the HIV virus. So her condition became worse.”* (Mother to deceased)

### Lack of family support during illness period

It was also evident in the study that there was poor care provided to the women at home. Caregivers reported that the deceased women were not supported by their immediate families and some ended up being hopeless. For instance, some women were not given food when they were sick, or it was reported that the women’s children were the caregivers. In one case, the woman did not want to go to hospital because she had to take care of her children since the husband would not do so because of cultural norms.

*“Another thing was lack of food, she used to take medicine and there was no food. So, we would take turns bringing her food, three of us, as she had nothing. It became hard and the chairman took her to hospital so that she could receive better care.”* (Friend to deceased)

*“She was treated at the nearby dispensary xxx. Due to insufficient supply of food, she was not able to cope as well as meet the family food needs since the children’s father was also not there.”* (Child to deceased, whose sex cannot be determined from the transcript)

*“Our daughter was married at xxx; she stayed there and gave birth to three children. The husband was staying far from her, but whether he comes or not life must continue. As years unfolded, she seemed to be getting older, the husband decided to marry another wife and that’s when he started neglecting her and problems started. She then decided to come back to bring her problems here.”* (Mother to deceased)

*“When she got ill the first time, the husband did not act fast enough to take her to hospital, and so the illness advanced too fast.”* (Neighbour to deceased)

## Discussion

The objective of this study was to explore the socio-cultural and health system factors affecting health-seeking behaviour of deceased women in Kenya who were living with HIV (WLHIV) at the time of death. Similar to previous studies conducted in Uganda, Democratic Republic of Congo and Thailand [31-34], our study findings showed that the majority of caregivers are family members. This demonstrates that families play a major role in caring and supporting persons living with HIV/AIDS. Socioeconomic status was found to be a significant determinant of utilization of health care services. In particular, a higher proportion of poor women did not have any contact with formal health services one month prior to death compared to women from non-poor households. These findings corroborate evidence from previous studies in developing countries which found that poverty is one of the key barriers to utilization of health services [15,17,20]. Although the Kenya government has over the years introduced various cost-reduction measures such as the introduction of waiver and exemption systems for HIV-related diseases, malaria and tuberculosis treatment and drugs, the implementation has been less successful. It is common for users to encounter some kind of fees for maternal and HIV and AIDS related health services and medication [35]. The findings further show that long distance and unaffordable travel costs were key barriers to clinic attendance and accessing care. Other studies on barriers to utilization of HIV-related services also found that distance to facility and transportation costs were critical barriers to accessing treatment [14,18,19,21].

In addition, lack of knowledge and poor understanding of signs and symptoms of AIDS-related illnesses were barriers to clinic attendance. Many of the women interpreted the symptoms as related to malaria or other common illnesses and, therefore, sought over-the-counter malaria medications. It also emerged that in some cases HIV/AIDS was associated with witchcraft and evil spirits. This led to delays in seeking care which can have negative effect on clinical outcomes among women living with HIV. It was also evident that women who perceived themselves as having low HIV risk were reported as having not sought HIV treatment. The finding on lack of knowledge as a barrier to utilization of HIV and STI health care services is consistent with those of similar studies in Brazil and India [36,13]. It is also consistent with the general trend in the country whereby despite near-universal awareness about HIV/AIDS among women and men aged 15–49 years, comprehensive knowledge of symptoms and ways of prevention is much lower [3].

Similar to other studies conducted in India and Tanzania, family and societal barriers were also cited as impediments to utilization of health services [13,23]. Stigma (both perceived self-stigma and experienced stigma) was blamed for non-disclosure of HIV status and this resulted in women delaying seeking care and treatment. Many caregivers reported that they learned about the women’s status from the health care providers. These findings were also observed in studies conducted in Zimbabwe, South Africa and Tanzania, where it was noted that there was need to consider the social context of HIV status disclosure, since the social context shapes the process of status disclosure especially with respect to the potential exposure to stigma [37-39]. HIV status disclosure is important, especially for expectant women since it can inform the provision of prevention of mother-to-child transmission (PMTCT) HIV services. Disclosure is also important for psychosocial support, treatment adherence and stigma reduction [40,41].

The delay in seeking medical care among women was largely influenced by lack of resources and the socially constructed role of women as the primary caregivers, especially for children. Women, therefore, preferred looking after their children to seeking medical care. The findings show that the individual risk of women and health seeking behaviours are influenced by wider contextual factors such as poverty, sexism and structural violence especially in rural Kenya where women are disproportionally affected with respect to access to wealth and power. Other studies found similar findings which demonstrated that family dynamics can be a barrier to utilization of health services [13]. Lack of family support, especially by men, was mentioned as a barrier to accessing treatment, with some, for example, insisting on spouses undertaking domestic chores even when they are ill. This led their spouses to decline seeking medical care and, more so, hospital admission in order to look after the children. This finding is consistent with evidence from previous studies, which demonstrated that male involvement is an important factor in facilitating women’s access to HIV-related services [42].

Health care system barriers including negative experiences with health care providers, poor referral systems, perceived lack of providers, lack of drugs and equipment as well as perceived poor quality of care were mentioned by caregivers as factors that discouraged women from seeking care from public health facilities. These factors have been cited by previous studies as contributors to irregular or delayed utilization of HIV-related services [13,14,16,18,21-23].

Although HIV testing is critical for HIV prevention programming, it was estimated that as of 2012, less than half of those infected with HIV in Kenya (47%) were aware of their status, which significantly limits opportunities for prevention, treatment and care [4]. However, there has been an enormous expansion of HIV testing and counselling (HTC) services in the country: the number of Voluntary Counselling and Testing (VCT) sites, for instance, increased from only three voluntary counseling and testing (VCT) sites established in government health facilities in 2000 to 4,438 sites by 2010 [43]. The increased integration of provider-initiated testing and counselling (PITC) into antenatal care services has resulted in more women being tested for HIV. Whereas integration of HIV testing into antenatal care is an important way to enhance access and ensure that HIV-positive women can fully exercise their reproductive rights, it is imperative that testing is accompanied by adequate counselling and respect for clients’ right to informed consent and confidentiality. As reported by caregivers in our study, perceived ‘coercive’ testing for HIV not only alienates women from the health care system, but also creates social distances between the women and the local community especially due to fear of disclosure of sero-status and stigma, despite the fact that the community is an important social capital required by the women. A national health workers survey conducted in Kenya in 2005, for instance, showed that 20% of health workers found it acceptable to test patients without their knowledge [44]. This calls for proper training of health workers on HIV testing and counselling guidelines and procedures.

Our study findings also showed that medical pluralism was a common practice among the women living with HIV. The majority of women living with HIV first opted for home treatment, then purchased over-the-counter drugs, then sought care from a traditional healer, and visited a health facility before going back to a traditional healer. Evidence from previous studies also showed a similar pattern [24]. Key determinants of medical pluralism were the level of perceived risk of HIV infection and severity of illnesses.

Another finding of the study is the significant association between medical pluralism on the one hand and age and marital status on the other. Specifically, older women were more likely to seek medical care from more than one source compared to their younger counterparts. Moreover, married women were more likely to seek care from more than one place compared to never married women. Previous research on medical pluralism in South Africa found similar patterns by age and marital status; however, the results were not statistically significant [45].

Although the performance of the vital registration system at the national level is poor, the fact that the study was able to identify deaths from the records kept by the local administration suggest that the system could be an important source of information on vital events if it is well managed. The chiefs maintain records of deaths occurring in their areas of jurisdiction and submit the data to the sub-county death and birth registration offices from where the data are sent to the national death and birth registration offices. However, at the national level, there are challenges with respect to data documentation and management because the vital registration system is not functioning well. There is therefore a need to improve management of the vital registration system at the national level in order to make it a source of quality and reliable data on vital statistics.

The findings of this paper may be influenced by the study’s limitations. Due to the fact that the respondents were the main caregivers of the deceased women, it was possible that the data may have been affected by different types of biases, including recall bias of past events and likelihood of providing socially desirable answers to sensitive questions. In addition, responses to verbal autopsy questions might have been influenced by the caregivers’ differential access to information about the women’s illnesses. There is a possibility that there were underlying differences in the responses provided by caregivers depending on the level of interaction with the health providers who attended to the women before they died. Moreover, societal attitudes and beliefs may not only affect discussions about death, but the extent to which HIV/AIDS-related issues are openly discussed by the caregivers as well. However, we were not able to account for these differences in our analyses. Due to financial constraints, there was no back-translation of the transcripts to local languages to determine if some meanings were lost during translation of the interviews into English.

## Conclusion

Universal access to HIV services for people living with HIV is a key agenda in the global and national debate in the fight against HIV/AIDS that seeks, among other things, to reduce HIV-related illnesses and deaths [46,47]. The findings of this paper highlight important issues that have implications for addressing challenges faced by women living with HIV, including non-adherence to treatment regimen and late diagnosis of HIV. Being poor, poor knowledge and lack of understanding of AIDS-related illness, long distance to facility and transportation costs, medical pluralism, stigma, perceived low risk of HIV infection, lack of family support and health care system barriers emerged as factors contributing to delays/constraints in seeking care. The factors cut across the different socio-economic groupings and regions of the country. In addition, the factors have negative consequences for patient outcomes and may, overall affect the effectiveness of national HIV care and treatment programs. HIV programs need to find ways of addressing these barriers in order to increase utilization of appropriate services. Although HIV treatment is free in public facilities, poor women are still not able to access health services. This could be because of other barriers such as costs associated with transportation and treatment for opportunistic infections. One recommendation is to include transportation subsidies as part of the national social safety-net strategy to address financial constraints associated with transportation costs among poor women living with HIV. In addition, proper training for health care providers and adequate supply of drugs and equipment in the public health systems is critical for addressing some of the challenges that are highlighted in the paper.

## Competing interests

The authors declare that they have no competing interests.

## Authors’ contributions

RN: Involved in the conceptual design of the study, data collection, data analysis, drafting, re-organizing and overall revision of the manuscript. JK: Involved in data analysis, drafting, re-organizing and overall revision of the manuscript. FO: Involved in data analysis and interpretation and revision of the manuscript. CW: Conceptual design of the study and revision of the manuscript. All authors read and approved the final manuscript.

## Pre-publication history

The pre-publication history for this paper can be accessed here:

http://www.biomedcentral.com/1472-6874/14/77/prepub
